# Social incentives improve deliberative but not procedural learning in older adults

**DOI:** 10.3389/fpsyg.2015.00430

**Published:** 2015-04-16

**Authors:** Marissa A. Gorlick, W. Todd Maddox

**Affiliations:** ^1^Department of Psychology, Yale UniversityNew Haven, CT, USA; ^2^Department of Psychology, University of Texas at AustinAustin, TX, USA; ^3^Institute for Mental Health Research, University of Texas at AustinAustin, TX, USA; ^4^Institute for Neuroscience, University of Texas at AustinAustin, TX, USA; ^5^Center for Perceptual Systems, University of Texas at AustinAustin, TX, USA

**Keywords:** age, social, feedback, learning, deliberative, procedural, automatic

## Abstract

Age-related deficits are seen across tasks where learning depends on asocial feedback processing, however plasticity has been observed in some of the same tasks in social contexts suggesting a novel way to attenuate deficits. Socioemotional selectivity theory suggests this plasticity is due to a deliberative motivational shift toward achieving well-being with age (*positivity effect*) that reverses when executive processes are limited (*negativity effect*). The present study examined the interaction of feedback valence (positive, negative) and social salience (emotional face feedback – happy; angry, asocial point feedback – gain; loss) on learning in a deliberative task that challenges executive processes and a procedural task that does not. We predict that angry face feedback will improve learning in a deliberative task when executive function is challenged. We tested two competing hypotheses regarding the interactive effects of deliberative emotional biases on automatic feedback processing: (1) If deliberative emotion regulation and automatic feedback are interactive we expect happy face feedback to improve learning and angry face feedback to impair learning in older adults because cognitive control is available. (2) If deliberative emotion regulation and automatic feedback are not interactive we predict that emotional face feedback will not improve procedural learning regardless of valence. Results demonstrate that older adults show persistent deficits relative to younger adults during procedural category learning suggesting that deliberative emotional biases do not interact with automatic feedback processing. Interestingly, a subgroup of older adults identified as potentially using deliberative strategies tended to learn as well as younger adults with angry relative to happy feedback, matching the pattern observed in the deliberative task. Results suggest that deliberative emotional biases can improve deliberative learning, but have no effect on procedural learning.

## Introduction

The demographics of the world are changing at a rapid pace and people are living and working longer than ever before. Late life participation in the workplace comes with an increased need to learn new skills as we age; however, our ability to use feedback to guide learning shows declines across the lifespan (Rhodes, 2004; Mell, 2009; Maddox et al., 2010; Simon et al., 2010a,b; Samanez-Larkin et al., 2012, 2014). Despite well-documented deficits in fluid cognition often associated with normal aging including memory, attention, speed of processing, and executive function (Park et al., 2002; Salthouse, 2004), crystalized intelligence and affective processing are generally well preserved (Blanchard-Fields et al., 1995; Leclerc and Kensinger, 2008; Mather, 2012) and older adults report enhanced emotional well-being compared with younger adults (Carstensen et al., 2000; Samanez-Larkin et al., 2014). *Socioemotional selectivity theory* (SST) posits that as the temporal horizons of mortality become more salient in older age, motivation shifts away from the pursuit of knowledge and information and toward an emphasis on satisfying social goals and maximizing emotional well-being (Fredrickson and Carstensen, 1990; Carstensen and Turk-Charles, 1994; Carstensen, 2006; Mather and Knight, 2006). Supporting this idea, older adults are better at solving problems with a social component (Blanchard-Fields et al., 1995) and are more likely to choose an emotionally gratifying familiar social partner relative to interesting but uncertain novel social partners (Fredrickson and Carstensen, 1990).

Age-related changes in motivation toward positive social experiences have been shown to influence cognition. In fact,* when cognitive tasks are rooted in positive emotionally salient contexts older adults demonstrate enhanced fluid cognition*. For example, in older compared to younger adults positive relative to negative emotional items receive more attentional resources (Isaacowitz et al., 2006; Knight et al., 2007) and are better remembered in working memory, long-term memory, and autobiographical memory tasks (Charles et al., 2003; Mather and Carstensen, 2003; Kennedy et al., 2004; Nielson and Powless, 2007). It has been demonstrated that this *age-related positivity effect* is the result of deliberative processes, or those that depend on executive function, that regulate emotional states to enhance well being and is not simply the serendipitous result of neural declines (Mather and Carstensen, 2005; Carstensen, 2006; Mather and Knight, 2006; Kryla-Lighthall and Mather, 2009). In support of this idea, the positivity bias disappears when executive resources are limited due to a secondary task (Isaacowitz et al., 2006; Knight et al., 2007), when participants are asked to seek threat (Mather and Knight, 2006), or when they are asked to weight all information equally (Löckenhoff and Carstensen, 2007). Thus, older adults deliberately allocate more resources to positive social experiences using cognitive control resources (Kryla-Lighthall and Mather, 2009). Importantly, when these resources are limited the positivity bias disappears and can sometimes reverse to become a negativity bias (Mather and Carstensen, 2005). Together this research reveals that declines in fluid cognition are more plastic than previously thought and goal-directed motivation to seek out positive information may attenuate well-established cognitive declines – particularly in socioemotional contexts.

**FIGURE 1:**
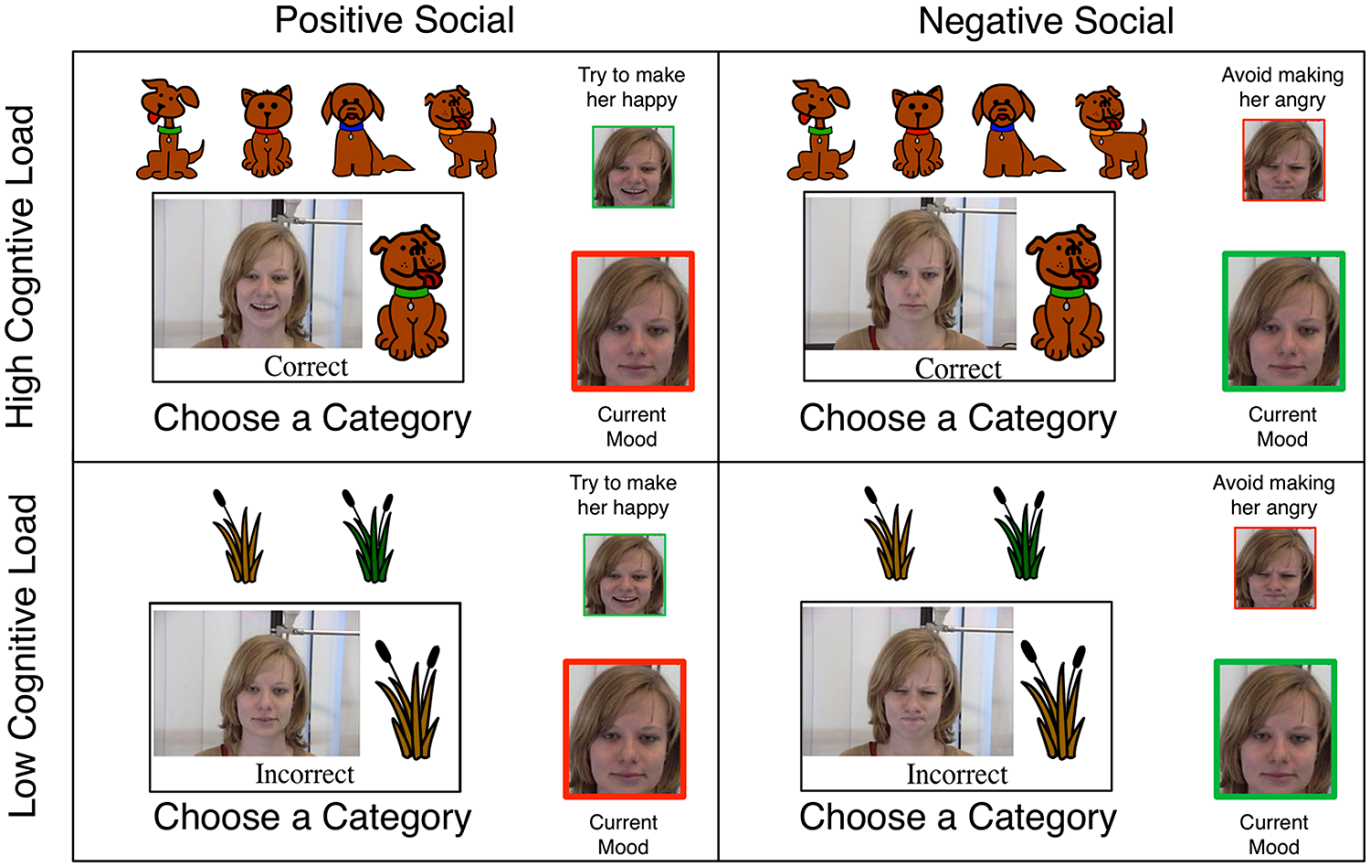
**An adapted schematic of the task from Gorlick et al. (2013)**. This task directly manipulated task demands on cognitive control. Behavior suggests that learning with social feedback depends on a complex three-way interaction between task demands (high cognitive control demand **(top)**: three dimensional stimuli each with four possible values, low cognitive control demand **(bottom)**: two dimensional stimuli each with two possible values), age (younger, older) and social feedback valence (positive happy face **(left)**, negative angry face **(right)**).

It is still an open question whether the cognitive plasticity associated with affective processing can be utilized to improve age differences in learning outcomes. Previous research examining deliberative category learning with set-shifting [Wisconsin Card Sort Task (WCST; Heaton et al., 1993)] demonstrates robust deficits in learning and increased perseverative errors in older adults (Rhodes, 2004). During the WCST explicit verbal rules are developed and elaborated upon using cognitive control which is mediated by the prefrontal cortex (Gunning-Dixon and Raz, 2003; Cappell et al., 2010). Here participants are presented with three dimensional exemplars and are asked to sort these items into one of four categories using “correct” and “error” feedback to guide learning. Unbeknownst to the participant, after ten consecutive correct responses the rule changes and cognitive control must be used to disengage from the first rule and adopt a new rule. Interestingly, recent research in our laboratory demonstrates that these cognitive deficits, which are considered a hallmark of age-related cognitive declines, can be attenuated when learning is placed in a social context (**Figure 1**; Gorlick et al., 2013). Importantly, the efficacy of social feedback depends on a complex three way interaction between task demands [high cognitive control demand (**Figure 1**, top): three dimensional stimuli each with four possible values, low cognitive control demand (**Figure 1**, bottom): two dimensional stimuli each with two possible values] social feedback valence [positive happy face (**Figure 1**, left), negative angry face (**Figure 1**, right)] and age (younger, older). When task demands on cognitive control were low, older adults demonstrated enhanced performance with positive, happy face feedback relative to negative, angry face feedback. However, when task demands on cognitive control increased, this pattern reversed and older adults demonstrated enhanced performance with negative, angry face feedback relative to positive, happy face feedback. Thus, social incentives can increase cognitive flexibility in older adults and can improve learning outcomes in deliberative tasks – but the valence of feedback and its interaction with task demands on cognitive control are critical in determining learning outcomes. Specifically, happy face feedback improves learning in tasks that place less demand on cognitive control and angry face feedback improves learning in tasks that place more demand on cognitive control.

Though this paradigm offers a potential method for attenuating age-related learning deficits without cognitive training or pharmaceutical interventions, it is still unclear how these findings extend to age-related deficits in automatic procedural category learning tasks. Rule-based task performance, such as that seen in the WCST and Gorlick et al. (2013), is considered deliberative because verbalizable rules are maintained by the central executive and used to guide performance (e.g., “categorize by color”). On the other hand, procedural information integration task performance is characterized by slow and incremental learning. The neural circuits underlying procedural strategies are largely striatal and critically do not utilize the prefrontal circuits that mediate deliberative executive processes (Ashby and Maddox, 2010). Here procedural processes develop knowledge of non-verbal mathematical relationships through corrective feedback (information integration categorization; Ashby and Maddox, 2010). Thus, accuracy is maximized if information from two or more stimuli dimensions is integrated at an automatic predecisional stage and not through an elaborate verbalizable strategy. Though the procedural and deliberative learning systems that excel at mapping different kinds category structures are mediated by dissociable neural systems, they are interactive both competing and cooperating to determine behavior (Poldrack and Packard, 2003; Poldrack and Foerde, 2008; Ashby and Maddox, 2010).

Research examining automatic learning systems have been mixed where tasks that do not utilize feedback processing are relatively intact across adulthood (perceptual representations, implicit pattern recognition; Howard et al., 2004a,b; Simon et al., 2010b; Glass et al., 2012), and other automatic learning systems that depend on feedback processing are impaired (procedural information integration; Filoteo and Maddox, 2004; Maddox et al., 2010). In tasks where feedback is used to guide learning, individuals tend to initially adopt a deliberative rule based strategy and may subsequently switch to automatic procedural strategies over time. Interestingly, older adults struggle to make the transition from deliberative to procedural approaches resulting in individual differences in the strategy employed during learning (Maddox et al., 2010). This suggests that individuals could implement deliberative or procedural strategies during the information integration task, which would likely have implications for performance.

Though it has been well established that deliberative and procedural learning systems are interactive, *it is still an open question whether this interactive systems framework extends to deliberative biases on automatic feedback processing*. An interactive feedback hypothesis might predict that deliberative emotion regulation strategies that enhance or attenuate deliberative feedback processing may also influence automatic feedback processing. In support of this idea, the lateral prefrontal cortex (lPFC) interacts with subcortical motivational structures to influence emotional experience during emotion regulation (Greening et al., 2014). One proposed account of the age-related positivity effect is that the lPFC expends executive resources to inhibit amygdala activation which decrease processing of negative emotional information and increase nucleus accumbens activation, a region of the striatum, which increases processing of positive emotional information (Wager et al., 2008; Winecoff et al., 2011; Greening et al., 2014). Importantly, because procedural category learning strategies are automatic, cognitive control resources would be available to facilitate a deliberative positivity effect, which in turn may tune automatic feedback processing through subcortical motivational structures. With this in mind, *one prediction is that happy face feedback will attenuate age deficits in procedural information integration learning and negative angry face feedback will accentuate these deficits*. However, it is also possible that deliberative and automatic feedback processing is not interactive. In fact, some research suggests procedural category learning depends on rapid, *automatic* feedback processing. Rich verbal feedback about category membership (Maddox et al., 2008) and delaying feedback (Maddox et al., 2003) increase the use of deliberative strategies and can impair procedural performance (Maddox and Ashby, 2004; Filoteo et al., 2010). Thus, *one alternative prediction states that deliberative social feedback processing will have no effect on procedural category learning.*

Together, we had two competing hypotheses about the way emotional feedback would influence procedural category learning. One hypothesis predicts that deliberative emotional biases interact with automatic feedback processing and when cognitive control resources are available during procedural learning older adult deficits can be attenuated with positive happy face feedback but not negative angry face feedback, however, an alternate hypothesis states that deliberative emotional biases do not interact with automatic feedback processing predicting that the deliberative positivity effect will only benefit deliberative learning resulting in pervasive age deficits in the procedural categorization task.

**FIGURE 2:**
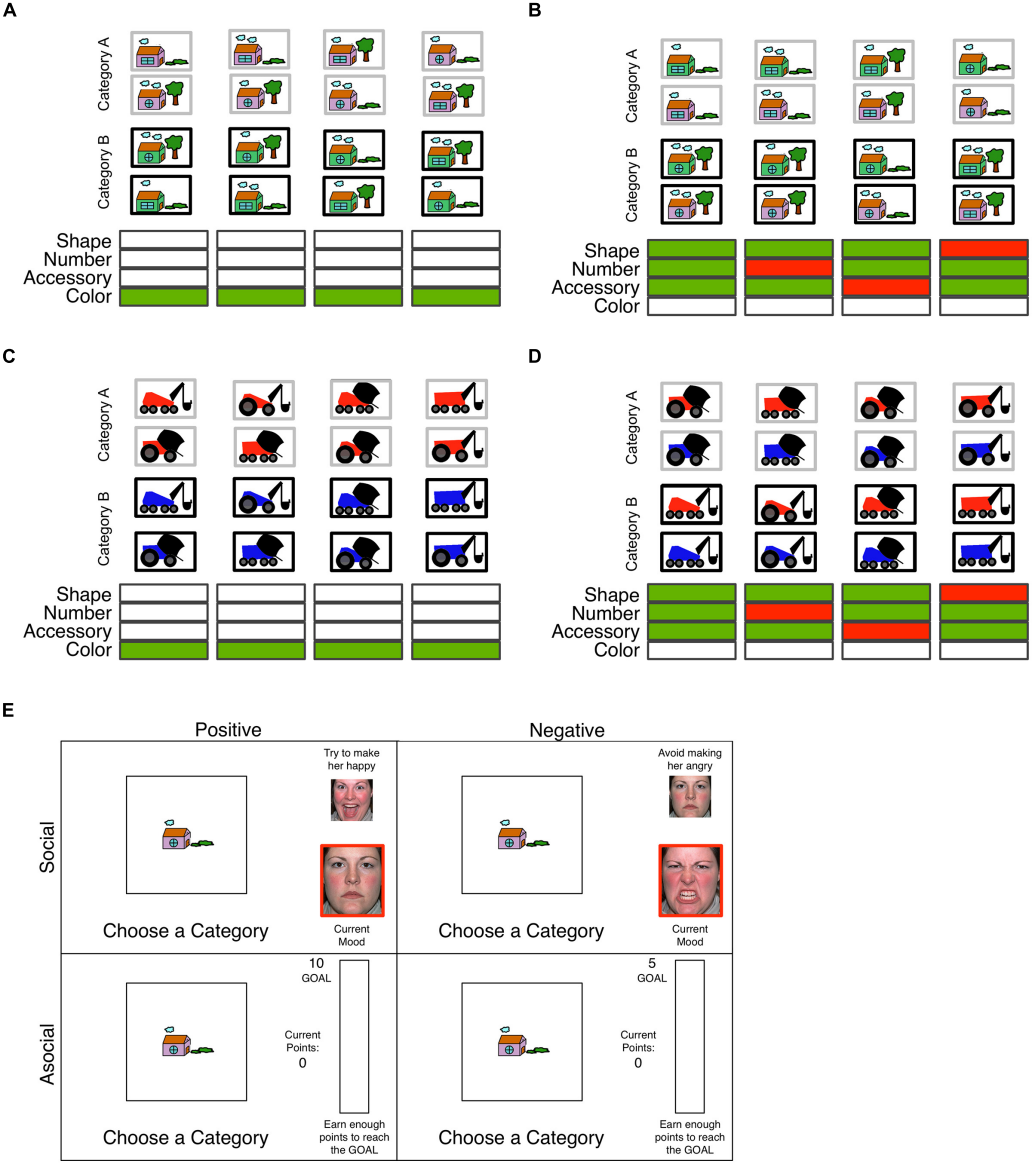
Example surface features (houses, vehicles) with an example of category membership for the rule-based (**A,C**; rule = color) and information integration (**B,D**; rule = shape (window, body), number (cloud, wheel) accessory (landscape, tool); see Materials and Methods) tasks. Features disambiguating category membership are highlighted in green below each column in green. **(E)** Screen shots for the social (top) and asocial (bottom) feedback manipulations. Within each of these condition the valence of the feedback was either positive (left; increase happiness or approach the goal) or negative (right; decrease anger or avoid increasing the goal).

To test these hypotheses, the present study explores how the valence of social feedback (happy faces, angry faces) and asocial feedback (point gains, point losses) differentially affects deliberative and automatic category learning in younger and older adults. The goals of this study are three fold. First, we test competing hypotheses about the effects of social feedback on age-related deficits in procedural category learning. To examine this question we asked participants to categorize complex 4 dimensional binary valued stimuli into one of two groups (see **Figures 2B,D**). If deliberative and automatic processes interact during feedback processing we expect to see an age-related advantage in the procedural information integration task given positive happy face feedback and an age-related impairment with negative angry face feedback. This result would be in line with other work that demonstrates a positivity bias when cognitive control resources are available (Knight et al., 2007; Kryla-Lighthall and Mather, 2009; Nashiro et al., 2011; Gorlick et al., 2013). However, if deliberative and automatic processes do not interact during feedback processing we expect to see no effect or age-related deficits with social feedback in procedural learning.

Second, we want to replicate and extend the findings from Gorlick et al. (2013) to a novel verbal rule-based task that is comparable to the procedural category learning task and prior work. To achieve this aim we examine a deliberative task with the same four dimensional binary valued stimuli as the procedural learning task (see **Figures 2A,C**). This task utilizes complex four dimensional stimuli that challenge cognitive control and working memory. As demonstrated in prior work (Waldron and Ashby, 2001), rule based category learning tasks like those in **Figures 2A,C** depend on cognitive control and performance is impaired under dual-task conditions. However, procedural information integration category learning tasks like those in **Figures 2B,D** do not depend on cognitive control and performance is not affected under dual task conditions (Waldron and Ashby, 2001). Thus, we expect to replicate the Gorlick et al. (2013) findings for the high cognitive load deliberative task in the current high cognitive load deliberative task (**Figures 2A,C**) whereby older adults showed no age-related learning deficit relative to younger adults with negative angry face feedback but did show an age-related learning deficit relative to younger adults with positive happy face feedback.

Finally, it is possible that emotional well-being can be achieved through asocial incentives. For example, individuals might be happy that they earned a monetary bonus or angry that they lost a monetary bonus, though monetary information has no inherent emotional content. Thus, it is possible that asocial incentives will improve learning outcomes in older adults in deliberative rule based and procedural information integration tasks as proposed above. In support of this idea, prior work has found that older adults have intact neural activation and subjective ratings of arousal during the anticipation of monetary gains but not monetary losses (Samanez-Larkin et al., 2007). However, the benefits of positive information in a non-learning context have not generalized to a task that requires learning (Gorlick et al., 2013; Samanez-Larkin et al., 2014). Thus, the final aim of the present work is to examine whether the social component of valenced feedback is critical in attenuating age-related learning deficits. To test this question we ran an asocial points condition on a small sample of individuals. As seen in prior work examining age differences in category learning in asocial contexts (Rhodes, 2004; Maddox et al., 2010; Gorlick et al., 2013), we predict a main effect of age across deliberative and procedural category learning tasks where older adults are less successful than younger adults.

## Materials and Methods

### Participants

Two hundred and eight participants (younger adults age 17–32, older adults age 60–88) were recruited to participate in the social face feedback conditions and 147 participants (younger adults age 18–36 and older adults age 57–88) were recruited to participate in the asocial point feedback conditions. As one of our hypotheses predicts that social face feedback may result in a null effect, we doubled our sample size in the information integration condition to increase power to detect an effect and avoid a type 2 error. There were no significant differences (all *p* > 0.05) between age nor years of education when comparing older adults or younger adults across valence conditions within each task (**Table 1**). Participants were offered either monetary compensation (older and younger) or course credit (younger only). The University of Texas at Austin Internal Review Board approved the procedures of this study and written consent was obtained for all participants.

**Table 1:** Sample size (*N*), Age in years, years of education (Bachelor’s = 16), and WAIS Vocabulary *Z*-Scores for **(A)** the social face feedback conditions and **(B)** Sample size (*N*), Age in years and years of education (Bachelor’s = 16) for the asocial point feedback conditions.

(A)
			*N*	Age	Education	Vocabulary
Information	Positive	Younger	37 (*F* = 21)	21.06 (3.52)	14.03 (1.84)	0.73 (0.99)
Integration		Older	37 (*F* = 21)	69.38 (5.89)	16.57 (3.18)	0.83 (1)
	Negative	Younger	37 (*F* = 23)	21.57 (3.44)	14.57 (2.01)	0.8 (0.82)
		Older	35 (*F* = 24)	70.11 (7.34)	16.91 (2.21)	0.83 (0.91)
Rule	Positive	Younger	17 (*F* = 8)	22.47 (4.73)	14.82 (2.24)	0.65 (1.09)
Based		Older	14 (*F* = 8)	66.43 (5.35)	17.07 (2.13)	1.15 (0.57)
	Negative	Younger	16 (*F* = 10)	21.81 (3.04)	14.94 (1.39)	0.58 (0.94)
		Older	15 (*F* = 6)	66.8 (6.19)	17.67 (2.35)	1.18 (0.83)
**(B)**
			***N***	**Age**	**Education**	

Information	Positive	Younger	20 (*F* = 12)	25.55 (5.81)	15.65 (1.69)	
Integration		Older	17 (*F* = 12)	65.94 (7.89)	16.12 (2.39)	
	Negative	Younger	20 (*F* = 13)	24.58 (4.9)	15.79 (2.32)	
		Older	22 (*F* = 14)	70.68 (6.66)	16.91 (3.31)	
Rule	Positive	Younger	20 (*F* = 11)	23.95 (5.08)	15.15 (1.31)	
Based		Older	12 (*F* = 7)	65.91 (8.56)	16.45 (2.42)	
	Negative	Younger	20 (*F* = 9)	24.45 (3.59)	15.85 (1.73)	
		Older	16 (*F* = 9)	72.12 (7.65)	15.69 (3)	

SD in parentheses.

In the social face feedback condition, older adults were administered a battery of neuropsychological tests (assessing attention, memory and executive function) to determine whether they were functioning within the normal range for their age. The neuropsychological battery includes the Wisconsin Card Sorting Test (Heaton et al., 1993), Wechsler Adult Intelligence Scale-Fourth Edition (Wechsler, 1981) Stroop test (Stroop, 1935), Trail-making test (Corrigan, 1987), and Wechsler Memory Scale (WMS-IV). All results were normalized for age using standardized procedures and converted to *Z*-scores. Neuropsychological tests were broken down into three subgroups of cognitive function. (1) Attention: Digit Span, (2) Memory: CVLT immediate and delayed, WMS-III Logical Memory immediate and delayed, and 3) Executive Function: Stroop Interference, Trails B, FAS, WCST perseverative errors. Older adults scoring 2 SD below the mean on more than one test from each of these subgroups were excluded (*N* = 1). Composite scores were generated for the three domains of fluid cognition (mean of *z*-scores) and compared across strategic groups for each participant. No significant differences emerged in attention, memory or executive function (all *p* > 0.05).

### Materials

In both the deliberative rule based and procedural information integration category learning tasks participants categorized four-dimensional, binary valued stimuli (a total of 16 possible exemplars) into one of two categories. The 16 possible exemplars were presented pseudorandomly (each of the 16 exemplars were randomly presented block-wise before repeating) until the goal was reached or 150 trials were completed.

Category membership was manipulated across tasks to examine learning within deliberative rule based and procedural information integration category learning systems. The procedural information integration category structure (II) was constructed as follows. First, one stimulus dimension was randomly selected to be made irrelevant. Then for each remaining stimulus dimension, the features were given a value of 1 or -1 and each stimuli’s category membership was determined by the following mathematical formula (where the three relevant stimulus dimensions are randomly assigned to X, Y, and Z): If X + Y + Z > 0; then category A; else category B. In the deliberative rule based task the optimal strategy was to apply a verbal rule-based strategy, whereas in the procedural information integration task the optimal strategy involved a predecisional integration of stimulus information (Grimm et al., 2008). In the deliberative rule based task the optimal strategy was to focus on one dimension in the face of interference from the three irrelevant dimensions to assign items with one level of the relevant dimension to category A and the other to category B. Though the current study uses a different category structure than Gorlick et al. (2013), this category structure has more features than both the high and low cognitive load tasks (two and three features, respectively), though it is somewhat less complex than the high cognitive load task where each exemplar was sorted into one of four categories (**Figures 1** and **2A,C**).

Two sets of surface features were used for category exemplars. In the house condition (**Figures 2A,B**), stimuli varied on the color of the house (green, pink), the shape of the window (circle, rectangle), the landscaping (tree, shrub), and the number of clouds (one, two). Participants were asked to classify each house into neighborhood A or neighborhood B. In the vehicle condition (**Figures 2C,D**), stimuli varied on the color of the vehicle (blue, red), the shape of the body (triangle, rectangle), the tool (mixer, excavator), and the number of wheels (two, four). Participants were asked to classify each vehicle into maker A or maker B.

### Procedure

The effects of feedback valence (positive, negative) on learning were examined in older and younger adults in four tasks that manipulated the social content of the feedback (socioemotional faces, asocial points) and category structure (rule based, information integration).

#### Social Feedback Task

Feedback valence (positive = happy faces, negative = angry faces) was manipulated between subjects (**Figure 2E**, upper panel). Feedback images were selected from a validated set of still images where a female model expresses happy, angry, and neutral emotions (Tottenham et al., 2009). The same model was used for both the happy and angry face feedback conditions. A spectrum of emotional intensity was created using MorphAge software that creates a controlled spectrum of emotion from highly arousing faces (either happy or angry) to neutral faces in steps of 10. In the positive social feedback condition, the participant’s goal was to make a face as happy as possible. Each correct response increased happiness by one step and each incorrect response returned the faces’ mood to neutral. After 10 consecutive correct responses the face reached its goal level of happiness and the task was complete. In the negative social feedback condition, participants must avoid making a face angry. Each correct response reduced anger by one step and each incorrect response returned the faces’ mood to the maximum amount of anger. After 10 consecutive correct responses the face was neutral and the task was complete. To ensure that female face feedback did not interact with the participant’s gender, participant gender was included in the analyses below (Results). No significant main effects or interactions with participant gender emerged.

#### Asocial Feedback Task

The asocial condition was similar to the emotion condition described above, however, here feedback is devoid of social content (**Figure 2E**, lower panel). Feedback valence (positive = approach goal, negative = avoid increasing goal) was manipulated between subjects. In the positive point feedback condition participants needed to earn 10 points to reach their goal. Each correct response increased their points by 1 and each incorrect answer dropped their points back to 0. After 10 consecutive correct responses the point meter was full and the task was complete. In the negative point version of the task, we manipulate avoidance behavior by starting the goal at 5 points and increasing the goal by 1 point with each incorrect response. These two approaches to reaching the goal have been demonstrated as an effective way to manipulate approach and avoid behavior (Grimm et al., 2008). Each correct response increased their points earned by 1 and each incorrect answer dropped their total points back to 0 and increased their goal by 1. Thus, in the negative point feedback condition participants avoid increasing their goal. In both tasks, their goal was reached when the point meter was full.

### Data Analysis

#### Performance

To examine performance differences within this task, we examined the number of trials needed to attain the goal. This is similar to the trials to learn the first rule examined in Gorlick et al. (2013) and has been used successfully in prior work to examine learning performance (Grimm et al., 2008). Notice that the goal in the positive point feedback condition and in the two social face feedback conditions is always 10 consecutive correct responses, whereas the goal in the negative point feedback condition could be something other than 10 consecutive correct responses in a row. In light of this fact, and in order to use the same dependent measure across the four conditions, we utilized the number of trials needed to reach 10 consecutive correct responses in a row in the negative point feedback condition even if that was not the relevant goal facing the participant at the time. No exclusions were made based on performance, though not everyone who completed the task reached the goal. Those who did not reach the goal were included in the analysis with trials to criterion set to 151 trials.

#### Strategy Analysis

Though the optimal strategy in the procedural information integration condition is to integrate information across dimensions, it is possible to meet the performance criterion of 10 in a row using a unidimensional rule if the set of randomly sampled stimuli is such that a unidimensional rule yields identical performance to the optimal strategy. Prior work has shown that older adults struggle to transition from a deliberative rule-based strategy to a procedural information integration strategy. Thus, it is likely that some individuals that completed the procedural information integration task did so with a deliberative rule based strategy. To examine the effects of strategy, we conducted a *post hoc* analysis grouping individuals by those who could have reached the goal by using a unidimensional rule (any of eight possible unidimensional rules could account for 10 consecutive correct responses across the last 10 trials as seen in the rule-based condition; II-UD) and those that used the optimal procedural information integration strategy for success during the last 10 trials (no unidimensional rules could account for 10 consecutive correct responses across the last 10 trials; II–II). Thus, those in the II-UD group may have been using a unidimensional rule-based strategy or an information integration strategy. On the other hand, those in the II–II group could not have been using a deliberative unidimensional rule and were more likely to be using a procedural information integration strategy. To anticipate, grouping participants by strategy will help us characterize nuances in the null effects seen in the procedural information integration task.

#### Executive Function

To test whether executive function was related to performance in the procedural information integration task and deliberative rule based task, we took a closer look at the older adult group in the social feedback task where we had rich information about executive function through the neuropsycholgical tests used to screen participants. A composite score for executive function was generated by taking the mean of the *z*-scores for all of the available measures of executive function within participants (Stroop Interference, FAS, Trails B and WCST perseverative errors). We correlated this executive function metric with the number of trials needed to reach the goal within each task across valence domains. We predicted that there would be no relationship with executive function in the procedural information integration task and a negative correlation in the deliberative rule based task where higher executive function scores would be associated with fewer trials needed to reach their goal.

## Results

### Social Face Feedback

#### Information Integration: Trials to Reach the Goal

**FIGURE 3:**
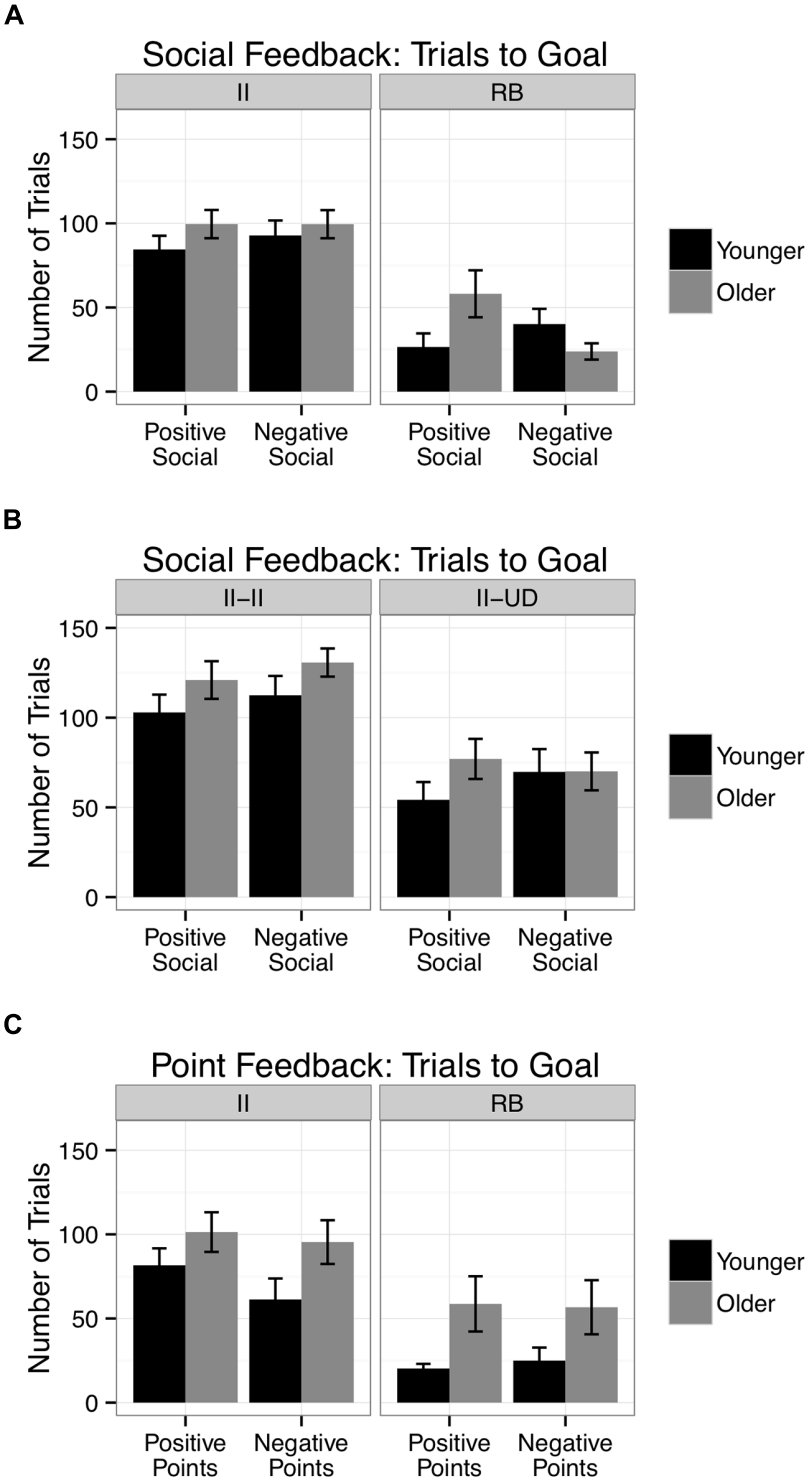
The number of trials needed to learn the rule for older and younger adults given **(A)** rule-based (RB) and information integration (II) category structures in the happy- and angry-face-feedback conditions **(B)** within the information integration condition by strategy (II-II = information integration strategy; II-UD = unidimensional strategy) and **(C)** the rule-based and information integration tasks in the point gain and point loss feedback conditions (II = information integration structure; RB = unidimensional structure). SE bars are included.

Within the information integration social feedback task, the number of trials needed to reach the goal was calculated for each participant and a 2 age (younger, older) × 2 valence (positive, negative) between subjects ANOVA was conducted (**Figure 3A**; left). There were no significant interactions, *F*(1,142) = 0.24, *p* = 0.62, η^2^ = 0.002, or main effects.

To ensure that the null effects seen in the social feedback information integration condition are not due to low power, we computed the Bayes Factor for older adults receiving positive happy face feedback and older adults receiving negative angry face feedback. These results support the null hypothesis, Scaled JZS Bayes Factor = 4.11, giving us confidence that there are no significant differences that went undetected (Rouder et al., 2009).

To ensure non-learners weren’t driving our results, we replicated these effects in those who reached the goal of 10 consecutive correct responses. age × valence interaction was non-significant, *F*(1,95) = 0.04, *p* = 0.85, η^2^ = 0.0004, and no other significant effects. There were no significant differences in the frequency of reaching the goal across valence by age group conditions, χ^2^(2, *N* = 146) = 1.03, *p* = 0.49.

To determine whether executive function was related to performance in the procedural information integration task, we correlated a composite score of executive function (see Materials and Methods) with the number of trials needed to reach the goal. There was no significant relationship between executive function and number of trials (*r^2^* = 0.01; *p* = 0.49) suggesting that executive processes are not associated with performance.

#### Information Integration: Strategy Use

To examine the influence of strategy use on performance in the information integration task, the data was divided into those that were using a procedural information integration strategy (II–II; **Figure 3B** left) and those possibly using a deliberative unidimensional rule-based strategies (II-UD; **Figure 3B** right; see Procedure; **Figure 3B**). When participants used the automatic procedural strategy in the information integration task (II-II), there was a marginal main effect of age *F*(1,75) = 3.26, *p* = 0.075, where older adults tended to require more trials to learn the rule relative to younger adults [*M*_Older_ = 125.56 (39.74); *M*_Younger_ = 107.35 (47.57)]. There were no significant effects of age or valence when participants may have used a unidimensional rule-based strategy during the information integration task (II-UD), however, there was a trend that matches the pattern in the rule-based task where older adults needed more trials than younger adults with positive happy face feedback [*M*_Older_ = 77.0 (47.38); *M*_Y ounger_ = 54.21 (37.03)] and performed similarly to younger adults with negative angry face feedback [*M*_Older_ = 70.05 (44.74); *M*_Y ounger_ = 69.76 (52.42)].

To test whether the application of the procedural information-integration strategy (II–II; Younger-Positive *N* = 23; Older-Positive *N* = 19; Younger-Negative *N* = 20, Older-Negative *N* = 17) or potentially deliberative unidimensional strategy (II-UD; Younger-Positive *N* = 14; Older-Positive *N* = 19; Younger-Negative *N* = 17, Older-Negative *N* = 18) strategy differed as a function of age and valence, we conducted a non-parametric chi-square test to compare the proportion of older and younger adults affiliated with each strategy. There was no effect of age or valence on strategy use, **χ**^2^(2, *N* = 147) = 1.4, *p* = 0.71.

### Rule Based: Trials to Reach the Goal

Within the rule-based social feedback task, the number of trials needed to achieve the goal was calculated for each participant and a 2 age × 2 feedback valence between subjects ANOVA was conducted (**Figure 3A**; right). There was a significant age × feedback valence interaction, *F*(1,58) = 6.58, *p* = 0.01, η^2^ = 0.10, that was characterized by older adults requiring more trials to learn the rule with positive happy face feedback relative to younger adults [*M*_Older_ = 58.14 (52.23); *M*_Younger_ = 26.53 (33.26)], *t*(29) = 2.05, *p* = 0.050. Older adults learned the rule with numerically fewer trials than younger adults with negative angry face feedback [*M*_Older_ = 23.87 (18.72); *M*_Y ounger_ = 40.12 (36.33)], *t*(29) = -1.55, *p* = 0.13, *ns*. Further, older adults needed more trials to learn the rule with positive happy face feedback than negative angry face feedback, *t*(27) = 2.39, *p* = 0.024. There were no other significant effects. These results replicate age-related biases for negative social feedback during an deliberative task that limits cognitive control resources (Gorlick et al., 2013).

To ensure non-learners weren’t driving our results, we replicated these effects in those who reached the goal of 10 consecutive correct responses. There was a significant age × valence interaction, *F*(1,58) = 8.74, *p* = 0.004, η^2^ = 0.14, and no other significant effects. There were no significant differences in the frequency of reaching the goal across valence by age group conditions, χ^2^(2, *N* = 62) = 1.03, *p* = 0.79.

To determine whether executive function was related to performance in the deliberative rule based task, we correlated a metric of executive function (see Materials and Methods) with the number of trials needed to reach the goal. There was a significant relationship between executive function and number of trials, *r^2^* = 0.14, *p* = 0.048, where greater executive function is associated with fewer trials needed to reach the goal suggesting that executive processes are being utilized to improve performance.

### Asocial Points Feedback

#### Information Integration: Trials to Reach the Goal

Within the information integration asocial task, the number of trials needed to reach the goal was calculated for each participant and a 2 age × 2 valence between subjects ANOVA was conducted (**Figure 3C**; left). There was a significant main effect of age, *F*(1,75) = 5.18, *p* = 0.03, η^2^ = 0.06, where older adults required more trials to learn the rule than younger adults [*M*_Older_ = 98.00 (55.29); *M*_Younger_ = 71.45 (51.11)], *t*(77) = 2.21, *p* = 0.03. There were no other significant effects.

#### Rule Based: Trials to Reach the Goal

Within the rule-based asocial task, the number of trials needed to reach the goal was calculated for each participant and a 2 age × 2 valence between subjects ANOVA was conducted (**Figure 3C**; right). There was a significant main effect of age, *F*(1,64) = 10.30, *p* = 0.002, η^2^ = 0.14, where older adults required more trials to learn the rule than younger adults [*M*_Older_ = 57.53 (60.10); *M*_Younger_ = 22.62 (25.74)], *t*(66) = 3.28, *p* = 0.002. There were no other significant effects.

## Discussion

When cognitive tasks are rooted in social contexts older adults often demonstrate remarkable plasticity in fluid cognitive abilities such as memory, attention and problem solving (Blanchard-Fields et al., 1995; Charles et al., 2003; Mather and Carstensen, 2003; Kennedy et al., 2004; Isaacowitz et al., 2006; Knight et al., 2007; Nielson and Powless, 2007). Recent research demonstrates that this plasticity extends to learning in deliberative categorization tasks that depend on generating verbal rules (Nashiro et al., 2011; Gorlick et al., 2013), however, the reach of these benefits during automatic procedural learning remain understudied.

The current study examined whether age-related deficits in automatic procedural learning can be attenuated using socially engaging faces as feedback. It is thought that the pursuit of social goals is facilitated by cognitive control resources that allow older adults to deliberatively enhance performance in positive social contexts. However when cognitive control resources are limited this positivity bias reverses to become a negativity bias enhancing performance in negative social contexts (Kryla-Lighthall and Mather, 2009). Further, though procedural learning is largely striatal, deliberative and procedural systems are interactive and have been demonstrated to both compete and cooperate to determine an optimal strategy (Poldrack and Packard, 2003; Poldrack and Foerde, 2008; Ashby and Maddox, 2010). However, it is unclear whether interactive deliberative emotional biases and automatic feedback processing also interact. Thus, we tested two competing hypotheses regarding how deliberative biases to pursue social goals might influence feedback processing in an automatic procedural task where cognitive control resources are available. One hypothesis is that deliberative emotional biases will interact with automatic feedback processing. Because cognitive control resources are available during procedural learning, we predicted that learning would improve with positive happy face feedback and learning would be impaired with negative angry face feedback in older adults. A second hypothesis is that deliberative emotional biases will not interact with automatic feedback processing. Under these conditions we predict deliberative emotional biases would have no effect on performance in a procedural information integration task.

In line with a hypothesis that states deliberative emotional biases are not interactive with automatic feedback processing, the results of this study demonstrate that emotional face feedback is ineffective at attenuating procedural learning deficits in older adults regardless of valence. These results are informative but should be interpreted with caution because a null effect in the information integration task does not provide definitive evidence that deliberative emotion regulation strategies do not interact with automatic feedback processes and deserve further study. Persistent procedural information integration learning deficits are in stark contrast to age-related enhancements in rule-based learning using negative emotional feedback when cognitive control is limited, replicating prior findings in a task similar to the Wisconsin Card Sort Task where executive function is limited (Gorlick et al., 2013). Importantly, though we see no age or valence effects in the information integration task, *post hoc* analyses indicate that strategy use may be an important factor in determining learning outcomes. Those older adults that used an information integration strategy in the procedural task (II–II) maintained deficits, however, those that may have been using a rule-based strategy (II-UD) demonstrate a trending interaction between valence and age as seen in the rule-based task where older adults are impaired relative to younger adults with happy face feedback and perform as well as younger adults with angry face feedback.

It is thought that enhanced attention for negative emotional information under limited cognitive control, as seen in the deliberative rule based task, may be due to automatic processes that seek unwanted thoughts and threats (Wegner, 1994; Mather and Knight, 2005). In our study, it is interesting to note that this would result in interactive deliberative feedback processing with automatic emotional biases in the rule-based task which is absent in the information integration task (automatic feedback processing with deliberative emotional biases) suggesting possible dissociations in the conditions under which we see automatic and deliberative processes interacting.

### Future Directions

Though our *post hoc* analysis of the strategies individuals are applying was a useful tool for understanding when we might see age deficits or enhancements, one limitation of this approach is that the II-UD group includes individuals that may have been using deliberative unidimensional rules or information integration strategies. In fact, it is possible that our marginal interaction in the II-UD group obscured by the inclusion of those using the information integration strategy. In addition, we do not see significant age deficits in the information integration task with social feedback or II–II strategy subgroup. Though the information integration analysis is confounded by strategic differences and the II–II strategic subgroup analysis is underpowered, it is possible that social feedback attenuates procedural learning deficits regardless of valence. Future work would do well to incorporate tasks where latent strategic differences can be better revealed using formal computational models (Ashby and Waldron, 1999; Maddox et al., 2007; Ashby, 2014). This could be achieved using stimuli with continuous valued dimensions where decision-bound models representing psychological category spaces can be applied to the data to more clearly estimate the nature of the strategy being used (deliberative or automatic; Ashby and Maddox, 1993). In addition, research examining age-related changes in strategy use and application are mixed. While some work indicates that older adults are more likely to rely on rule-based strategies (Mata et al., 2012), simple heuristics (Worthy and Maddox, 2012), and do so more consistently (Maddox et al., 2010) other work indicates that older adults are more likely to apply automatic strategies (Seaman et al., 2014). Thus, the precise conditions under which older adults favor deliberative and automatic strategies and how they interact with feedback valence are unclear and deserve further study.

Valenced information has also been implicated in differences in attention (Gorlick and Maddox, 2013; Zivot et al., 2013). Several studies have found that mood can modulate attentional weights where negative mood narrows attention and positive mood broadens attention in younger adults. These changes have been shown to influence strategy selection where narrow attention facilitates selection of unidimensional rules and broad attention facilitates information integration across features (however, see Gable and Harmon-Jones, 2008), however it is unclear how this valence by attentional focus interaction changes across adulthood and the implications for learning. Future work should better characterize the effects of valence, mood and attention on dissociable learning systems across adulthood.

In addition to age differences in motivation, other work indicates that individual differences in approach and avoid behavior can interact with feedback to influence task-directed motivation, or the ability to utilize task appropriate strategies (Grimm et al., 2008; Maddox et al., 2015). When global individual differences in approach/avoid and local motivational state are aligned (approach gains or avoid losses) cognitive flexibility is enhanced improving performance on deliberative tasks. However, when global and local motivational states are misaligned (approach losses or avoid gains) cognitive flexibility is reduced impairing performance on deliberative tasks. To date little research has considered age differences in conjunction with approach and avoidance global motivational traits. Interestingly, the positivity effect has been demonstrated as both increased attention and memory for positive items (approach positive) and decreased attention and memory for negative items (avoid negative) relative to younger adults indicating that individual differences and/or task context may influence the locus of these effects. However, it is still unclear how individual differences in trait approach and avoid behavior contribute to these processes (Mather and Carstensen, 2005; Maddox et al., 2015). Future work should better characterize individual differences in approach and avoidance to develop a more comprehensive picture of the motivation cognition interface.

## Conclusion

Age-related improvements in learning with negative angry face feedback generalize across deliberative verbal tasks that challenge cognitive control, however, cognitive plasticity related to emotional biases does not extend to procedural category learning. The null effects in the information integration task highlight the importance of considering strategy use in addition to manipulating category structures to develop a complete understanding of the factors driving performance in dissociable learning systems. Supporting evidence from Gorlick et al. (2013), the current work also demonstrates that the social component of feedback is critical in driving valenced feedback effects during deliberative learning. In the asocial point feedback task older adults demonstrated deficits classically seen in both rule-based and procedural tasks (Rhodes, 2004; Maddox et al., 2010; Gorlick et al., 2013). Thus, we are more confident that the social content of feedback is critical in reducing deliberative learning deficits in older adults.

## Conflict of Interest Statement

The authors declare that the research was conducted in the absence of any commercial or financial relationships that could be construed as a potential conflict of interest.
